# Influence of antecedent soil moisture content and land use on the surface runoff response to heavy rainfall simulation experiments investigated in Alpine catchments

**DOI:** 10.1016/j.heliyon.2023.e18597

**Published:** 2023-07-22

**Authors:** Gertraud Meißl, Klaus Klebinder, Thomas Zieher, Veronika Lechner, Bernhard Kohl, Gerhard Markart

**Affiliations:** a Department of Geography, University of Innsbruck, Austria; b Department for Natural Hazards, Austrian Research Centre for Forests (BFW), Innsbruck, Austria

**Keywords:** Repeated rainfall simulation experiments, Antecedent soil moisture content, Surface runoff generation, High precipitation intensity, Alpine catchments, Porosity

## Abstract

In small Alpine catchments, floods are mostly triggered by surface runoff generation during convective heavy precipitation events. Their magnitude also depends on the antecedent soil moisture content, which was shown in several previous studies. This study aims at understanding (a) which sites change their surface runoff response to rainfall events with high precipitation intensity under very moist pre-conditions to what extent and (b) on which site characteristics this depends on. Therefore, we conducted repeated rainfall simulation experiments (40–80 m^2^, 1 h, 100 mm h^−1^) at 20 sites in five Eastern Alpine areas and analyzed their results on the basis of soil-physical parameters derived from collected soil samples.

The hay meadow sites reacted with a strong increase in surface runoff to reduced saturation deficits, the pasture sites showed a smaller but visible response. The forest sites had the highest water retention capacities. The change in the surface runoff response is a function of the saturation deficit at the beginning of the initial experiment (r = −0.58). The soil physical parameters, especially the fine pore fraction (r = 0.56), correlate with the difference of the total surface runoff coefficient between the initial and the repeated experiment. The fine pore fraction also shows a high correlation (r = −0.78) with the saturation deficit at the beginning of the initial experiment, although pores of this fraction were saturated during all experiments. (Non-quantifiable) Land use effects, which in turn influence the soil physical parameters, play an important role in explaining how the surface runoff response in the repeated rainfall simulation experiment differs from the initial experiment. The information on land use and soil characteristics allowed the sites to be categorized into four types in terms of surface runoff disposition and the increase in total surface runoff coefficient in the second rainfall simulation experiment.

## 1 Introduction

Flood events such as the one See in Paznaun and Sellrain (Tyrol, Austria) in June 2015 are linked to precipitation events with extreme precipitation intensities and/or totals. Frequently, as was the case in June 2015 [1,2], they are also associated with increased antecedent soil moisture content (ASMC). ASMC strongly influences runoff formation at the plot as well as the catchment scale, especially surface runoff [3], which plays a significant role in mountainous catchments and may be responsible for flash floods causing great damage in a short time without warning.

Already Western and Grayson [4] reported on a threshold-like dependence of surface runoff on the wetness conditions in an Australian catchment (0.11 km^2^) with abruptly increasing runoff coefficients above a particular soil moisture content. Similar results were obtained by Latron and Gallart [[5], [6]] in a Mediterranean catchment (0.56 km^2^) as well as by Penna et al. [7] in a 1,9 km^2^ sized Alpine catchment. Meißl et al. [8] analyzed in an Alpine catchment (size 9.2 km^2^) the saturation deficit relating the ASMC to the respective pore volume and concluded that exceptionally high runoff coefficients occurred only in case of a saturation deficit lower than 0.28 (i.e., 72% of pore volume filled, mean value for the catchment). The increase in surface runoff with increasing ASMC can be attributed to several reasons: If overland flow is due to saturation excess, a higher ASMC means a lower unsaturated pore volume and thus results in a higher runoff coefficient. The size of the saturated area can also vary over time depending on ASMC [9,10]. If, on the other hand, infiltration excess is the cause of surface runoff, higher soil moisture content can lead to a reduction in infiltration capacity [10]. In particular, the increase in soil moisture can lead to swelling processes and thus to the closure of shrinkage cracks, as Zehe et al. [11] observed in a small catchment in the headwaters of the Danube using small-plot rainfall simulation experiments. On water repellent soils, however, low ASMC leads to strongly enhanced surface runoff [12,13], when hydrophilic switch to hydrophobic conditions [11].

The influence of soil moisture on the development of surface runoff for extreme precipitation events has been investigated with the help of modelling approaches (e.g. Ref. [[14], [15]]). However, experimental investigations are necessary in order to ensure that the models correctly represent the respective processes, and because extreme precipitation events are difficult to observe in the field. In this context, heavy rainfall simulation experiments have proven their worth [[16], [17], [18], [19], [20], [21], [22], [23]]. In order to produce representative results and, for example, to reduce the influence of lateral runoff from the area or of too short flow paths within the plot, the plot should be at least 40 m^2^ in size [18,[24], [25], [26], [27], [28], [29]]. There is not much research on the necessary plot length. Sadeghi et al. [30] experimented with lengths between 2 and 25 m and stated that the appropriate slope length should correspond with the slope lengths in the catchment. However, they investigated small catchments (1 ha) with short slope lengths.

Large-plot rainfall simulations were conducted in two elaborate studies, which focused, among other subjects, on the role of antecedent rainfalls for runoff generation:

• Scherrer [22] and Scherrer et al. [23] irrigated 18 plots (60 m^2^, 4 m × 15 m) on meadows, pastures and in forests in Switzerland from the Jura to the Alps with a precipitation intensity of more than 50 mm h^−1^ for several hours. On 11 plots, one of them at an Alpine meadow, they repeated the rainfall simulation experiment the next day. They found that it depended on the runoff generation process if antecedent rainfall had an impact on the runoff volume. Plots with infiltration excess and fast saturation excess overland flow reacted rapidly anyway, thus the runoff volume was independent of the actual soil moisture content. On sites, where the runoff response was dominated by slow saturation excess overland flow or by subsurface flow, the nature of the macropore system determined whether the site was able to drain overnight and consequently how much the first irrigation influenced the initial conditions and the outcome of the second experiment.
• Ries et al. [21] conducted a series of irrigation experiments at 23 sites (meadows and fields, 100 m^2^, 10 m × 10 m) in Baden-Württemberg (Germany). The first two experiments were dedicated to the analysis of the effect of antecedent soil moisture. For this purpose, a 60-min irrigation with a rainfall intensity of a return period of 100 years was carried out and repeated the following day. With the exception of five meadow sites, all experimental sites showed a clear difference in runoff formation between the first and second experiment. The volume runoff coefficient increased significantly in the second experiment, especially on the arable land. Meadow sites, on the other hand, showed a greater variability in runoff formation between the individual sites. This was also attributed to the properties of the macropore system, which - in contrast to the arable land - can form without ploughing.

Both studies were conducted in foothills and low mountain ranges (with the exception of one plot in the Swiss Alps). However, Alpine soils differ from extra-Alpine soils in the high spatial variability of their properties. This is due to the high variability of relevant soil forming factors such as climate, geology, relief and vegetation [30]. Among these factors, the characteristics of the relief with the corresponding morphodynamic processes play a special role in terms of redistribution of geological substrates, local water budget and microclimatic differences on a small scale [31]. This results in a small-scale variability of the soils and their properties. Based on these specific soil forming conditions, Alpine soils are often shallow and with a high proportion of coarse fragments [32]. As these soil characteristics affect the runoff generation and hence, the formation of floods, it is necessary to investigate runoff formation at Alpine sites.

Therefore, the goal of this study is to investigate the influence of ASMC and the saturation deficit, respectively, on the runoff behaviour of Alpine sites. The focus is laid on surface runoff, which is responsible for floods in steep torrential catchments [19,[33], [34], [35]]. Our research questions are:

• Which sites change their surface runoff response under very moist pre-conditions and – if they do so – to what extent?
• Which site characteristics does this depend on?

We conducted repeated heavy rainfall simulation experiments at 20 plots (hay meadows, pastures, forests, openings) in the Eastern European Alps, did supplementary soil physical analyses and used statistical analysis to quantify site characteristic effects on the ASMC influence on surface runoff generation.

## 2 Material and methods

### 2.1 Study areas

**Fig. 1:**
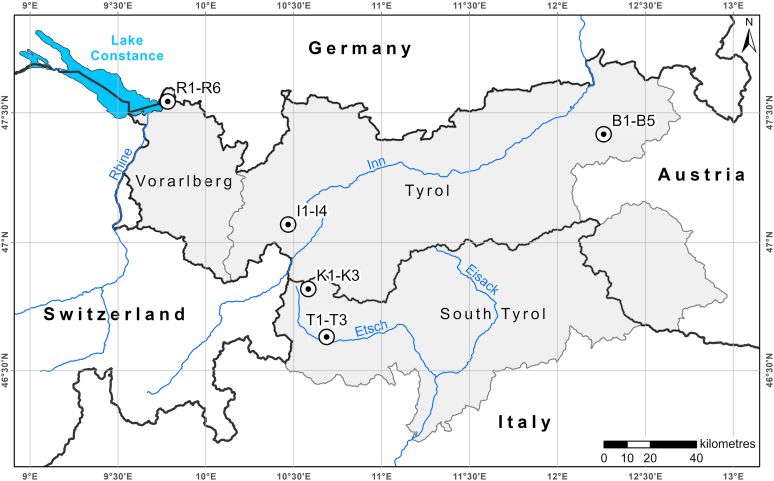
Locations of the rainfall simulation experiments (ESRI basemap). R1-R6 sites in the Ruggbach catchment, B1–B5 sites in the Brixenbach catchment, I1–I4 sites in the Istalanzbach catchment, K1–K3 sites in Kapron, T1-T3 sites in Tanas.

The rainfall simulation experiments took place in five areas in the Eastern Alps (Fig. 1). The experiments were conducted in two research projects focusing on the runoff generation, considering varying system states [36] as well as land cover and management impact [37,38], respectively. Three of the test areas are well defined hydrological catchments (Ruggbach, Brixenbach, Istalanzbach - Austria), two study areas lie on valley flanks on more or less stretched slopes, crossed by avalanche tracks and gullies (Kapron, Tanas - Italy). Table 1 shows the most important characteristics of the irrigation plots. They have four land use types: (a) pastures grazed by cattle from May to September, (b) hay meadows mown several times a year for forage and possibly grazed briefly in autumn, (c) forests, (d) openings within forests that have been cleared of forest due to forestry use (I1, I4) or avalanches (K2). Table 2 gives the mean annual precipitation s of the test sites. Moving from the north rim of the Alps (Ruggbach) to the inner Alpine dry valley of the Vinschgau (Tanas), the annual precipitation decreases significantly.

**Table 1:** Characteristics of the test plots.

Code	Longitude (East)	Latitude (North)	Geology	Soil	Land use	Soil profile depth [cm]	Ground cover by vegetation [%]
Ruggbach catchment							
R1	9°46′53.54″	47°32′18.64″	till on upper freshwater molasse	Cambisol	hay meadow	50	99
R2	9°46′58.27″	47°32′44.27″	upper freshwater molasse	Cambisol	hay meadow	40	99
R3	9°47′19.7″	47°32′49.94″	upper freshwater molasse	Cambisol	hay meadow	30	99
R4	9°47′1.63″	47°32′21.29″	upper freshwater molasse	Cambisol	forest (mixed)	50	5
R5	9°46′59.78″	47°32′20.05″	upper freshwater molasse	Gleyic Cambisol	forest (mixed)	50	75
R6	9°46′47.8″	47°33′1.05″	upper freshwater molasse	Cambisol	hay meadow	70	80
Brixenbach catchment							
B1	12°15′47.37″	47°25′27.93″	porphyroid	Cambisol	pasture	60	99
B2	12°15′23.29″	47°24′32.87″	till on clastic metasediments	Cambisol	pasture	40	99
B3	12°16′14.17″	47°25′20.09″	till on clastic metasediments	Stagnosol	pasture	40	99
B4/B5	12°16′12.84″	47°25′33.62″	till on clastic metasediments	Leptosol	pasture	40	80
Istalanzbach catchment							
I1	10°28′22.87″	47°4′8.1″	till on micaschists/paragneisses	Cambisol	opening	50	90
I2	10°28‘22.92″	47°4‘7.3″	till on micaschists/paragneisses	Cambisol	forest (coniferous)	50	50
I3	10°28‘22.25″	47°4‘6.79″	till on micaschists/paragneisses	Cambisol	forest (coniferous)	55	50
I4	10°27‘49.73″	47°4‘7.37″	till on micaschists/paragneisses	Podzol	opening	50	0
Kapron							
K1	10°34‘54.78″	46°49‘5.55″	till on micaschists/paragneisses	Cambisol	forest (coniferous)	40	99
K2	10°34‘56.53″	46°49‘6.32″	till on micaschists/paragneisses	Leptosol	opening	50	0
K3	10°35‘0.88″	46°49‘7.85″	till on micaschists/paragneisses	Cambisol	forest (coniferous)	60	80
Tanas							
T1	10°40’4.56″	46°40‘4.09″	orthogneiss	Cambisol	pasture	60	0
T2	10°40’5.10″	46°40‘4.0″	orthogneiss	Cambisol	forest (coniferous)	60	90
T3	10°40’6.78″	46°40‘2.21″	orthogneiss	Cambisol	forest (mixed)	60	99

**Table 2:** Mean annual precipitation measured at precipitation gauges near the test sites. Data sources: Austria: ehyd.gv.at, period 1991–2018, Italy: https://wetter.provinz.bz.it/download-messdaten.asp 1991–2020 (Eyrs – Laas due to a rain gauge transfer only 2010–2022).

Test area	Country	Nearby precipitation gauge	Altitude a.s.l. [m]	Latitude (East)	Longitude (North)	Mean annual precipitation [mm]
Ruggbach	Austria	Hörbranz	447	09° 45’ 10″	47° 33′ 01″	1509
Brixenbach	Austria	Aschau	1005	12° 18′ 29″	47° 23′ 00″	1502
Istalanzbach	Austria	See im Paznaun	1040	10° 27′ 53″	47° 05′ 03″	1009
Kapron	Italy	Melag	1915	10° 39′ 24″	46° 50′ 15″	790
Tanas	Italy	Eyrs - Laas	874	10° 39′ 00″	46° 37′ 29″	603

### 2.2 Measurements

**Fig. 2:**
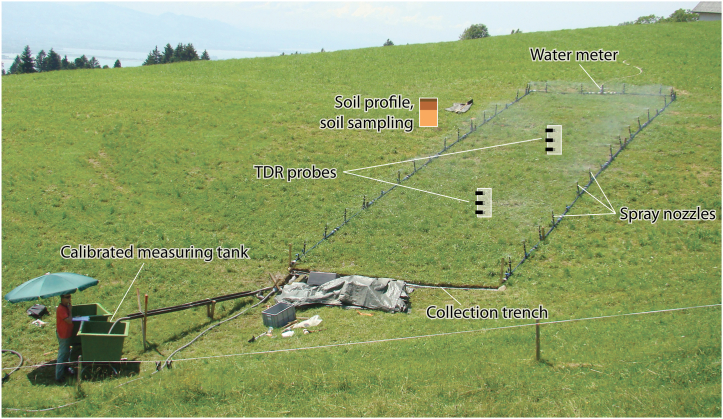
Large plot rainfall simulator at site R6 (Ruggbach). Photo: Klaus Klebinder.

We used a transportable rainfall simulator for large plots (see also Ref. [27]). It is 5 m wide and has a variable length up to 20 m (Fig. 2). We irrigated plot sizes between 40 and 80 m^2^ depending on limitations regarding topography and water availability. The rainfall simulator consists of quadrant (0.18 m^3^ s^−1^ in the edges and along the upper boundary) and semi-circle commercial nozzles (0.36 m^3^ s^−1^ along the downward sides of the plot) in order to cover the test plot as well as possible (sprinkling distance 4.8 m). Taking into account the spray pattern of the nozzles, the Christiansen Uniformity Index is 80% for an area of 50 m^2^. Water is supplied by stream water, pumped with a fuel-operated water pump, or by fire brigade hydrants. Water input is controlled by an automatically registering water meter (pressure gauge). At the lower end of the irrigated area, a trench collects the resulting surface runoff and leads it to calibrated measuring tanks with water level markings every 10 l [18,35]. The tank filling is observed by a person, who notes at which time the water level markings are reached. In order to guide the water into the trench, metal plates covered by a plastic sheet are placed 5–10 cm below soil surface above the trench. Thus, the measured discharge also comprises some near surface runoff.

We used time domain reflectometry (TDR) sensors (SOILMOISTURE® buryable waveguides) to measure soil moisture contents before, during and after the rainfall simulation in two profiles with one sensor each at 5 cm, 15 cm and 25 cm depth located in the centre of the experimental plot. Due to the high gravel content and the small-scale spatial variability of Alpine soils, we could not enhance the measurement accuracy by calibration and thus used uncalibrated values. The soil moisture values can therefore be compared very well within a site over the course of time of the successive experiments. When comparing between sites, the somewhat higher uncertainty of the measured values must also be taken into account.

We aimed at applying a sprinkling intensity of 100 mm h^−1^ with a usual experiment duration of 60 min, which is higher than that of a precipitation event with a return period of 100 years in the study areas, but allows to compare the results with several hundreds of previous experiments [16,35,[39], [40], [41]].

Table 3 shows the plot size, dates and duration of the rainfall simulation experiments. The course of the experiments was adapted to the experience gained, the site conditions (e.g., regarding the water supply) and the respective project aims. At the beginning of the first project, within which the rainfall simulation experiments presented here were conducted, the following procedure was executed: After setting up the sprinkler system, a first rainfall simulation experiment was carried out under the actual soil moisture content. Subsequently, at least another 100 mm were applied to the sprinkling plot for approx. 1–1.5 h (without measurement) in order to saturate the soil as much as possible. Finally, a second rainfall simulation experiment was conducted under conditions with high antecedent soil moisture content. This scheme was applied at the sites B1, B3 and R1. However, we found that during the unmeasured intermediate irrigation phenomena worth measuring occurred, e.g., a rapid increase in surface runoff. Therefore, surface runoff was subsequently measured during each irrigation. In this way, two to three experiments were carried out per site. For the rainfall simulation experiments conducted within the second project in 2018 (Istalanzbach, Kapron, Tanas), the duration of the second experiment was reduced to 30 min as a quasi-steady runoff was reached within this time at each plot.

**Table 3:** Rainfall simulation experiments. Notes: (1) Between the first and second experiment, a total of 96 mm was irrigated for 67 min. (2) Between the first and second experiment, there were 20 days and natural rainfall events. Between the second and third experiment, a total of 115 mm was irrigated for 1.5 h. (3) At this site, the experiments were not carried out on the same plot, as is usually the case, but on two adjacent plots, on a convex slope section (rather dry) and in a humid depression. (4) Between the first and second experiment, a total of 160 mm was irrigated for 1.5 h. (5) Before the grazing season. (6) Same plot as B4, after the grazing season.

Code	Plot size [m^2^]	Plot length [m]	Plot slope [°]	Date and duration		
				Experiment 1	Experiment 2	Experiment 3
R1^(1)^	80	16	14	August 23, 2011 – 60 min	August 23, 2011 – 50 min	–
R2	60	12	15	August 25, 2011 – 60 min	August 25, 2011 – 60 min	August 25, 2011 – 60 min
R3	80	16	15	July 02, 2012 – 60 min	July 02, 2012 – 60 min	July 02, 2012 – 30 min
R4	80	16	30	July 03, 2012 – 60 min	July 03, 2012 – 60 min	–
R5	60	12	34	July 04, 2012 – 60 min	July 04, 2012 – 60 min	–
R6	80	16	15	July 05, 2012 – 60 min	July 05, 2012 – 60 min	–
B1^(2)^	40	8	31	July 06, 2011 – 60 min	July 26, 2011 – 60 min	July 26, 2011 – 60 min
B2^(3)^	50	10	25	July 12, 2011 – 60 min	July 12, 2011 – 60 min	–
B3^4)^	40	8	15	July 27, 2011 – 60 min	July 27, 2011 – 60 min	–
B4^5)^	80	16	29	May 31, 2012 – 48 min	May 31, 2012 – 60 min	–
B5^6)^	80	16	29	September 04, 2012 – 60 min	September 04, 2012 – 60 min	–
I1	50	16	36	August 06, 2018 – 60 min	August 06, 2018 – 25 min	–
I2	50	10	36	August 07, 2018 – 60 min	August 07, 2018 – 20 min	–
I3	50	10	17	August 07, 2018 – 60 min	August 07, 2018 – 25 min	–
I4	50	10	34	August 09, 2018 – 60 min	August 09, 2018 – 25 min	–
K1	50	10	35	July 16, 2018 – 60 min	July 16, 2018 – 30 min	–
K2	50	10	11	July 17, 2018 – 60 min	July 17, 2018 – 30 min	–
K3	50	10	11	July 17, 2018 – 60 min	July 17, 2018 – 30 min	–
T1	50	10	21	July 18, 2018 – 60 min	July 18, 2018 – 30 min	–
T2	50	10	19	July 19, 2018 – 60 min	July 19, 2018 – 40 min	–
T3	50	10	11	July 20, 2018 – 60 min	July 20, 2018 – 30 min	–

Several characteristics of the resulting surface runoff hydrographs (SRH) were derived by fitting a constrained B-spline using the package ‘cobs’ of the R statistical programming language [42,43]. The parameters for fitting the spline were adjusted to the specific characteristics of the discharge curves at each location. In general, the spline was forced to start with the onset of surface runoff. Furthermore, for fitting the spline higher weight was given to the initial phase of the experiments' records, in order to better reproduce the initial increase and subsequent transition to rather constant surface runoff coefficient (SRC) values. Starting with a value of 0.9, the considered weights were reduced linearly down to a value of 0.5.

**Fig. 3:**
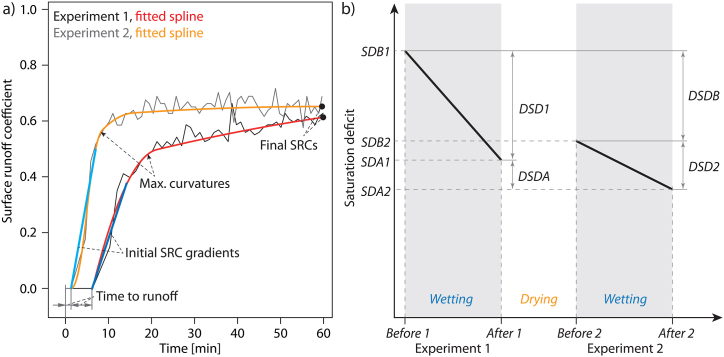
(a) Parameter definition shown on the example of the surface runoff hydrograph of the experiments at site B2, SRC = surface runoff coefficient, (b) typical development of saturation deficits during the experiments: SDB1 saturation deficit before experiment 1, SDA1 saturation deficit after experiment 1, SDB2 saturation deficit before experiment 2, SDA2 saturation deficit after experiment 2, DSDB saturation deficit before experiment 2 minus saturation deficit before experiment 1, DSDA saturation deficit after experiment 2 minus saturation deficit after experiment 1, DSD1 saturation deficit before experiment 1 minus saturation deficit after experiment 1, DSD2 saturation deficit before experiment 2 minus saturation deficit after experiment 2 (see also Table 6a).

The following SRH parameters were used to evaluate the rainfall simulation experiments (Fig. 3):

(1) Total SRC: surface runoff coefficient of the rainfall simulation experiment, calculated as quotient of the total surface runoff and total precipitation. It describes the runoff response very well, but is dependent on the experiment's duration, which must be taken into account in the experiments that lasted shorter than 60 min.
(2) Final SRC: final surface runoff coefficient, i.e., surface runoff coefficient derived from the fitted spline at the end of the rainfall simulation experiment. It is particularly meaningful for those experiments in which a quasi-steady runoff was achieved at the end of the experiment.
(3) Time to runoff: time span between the start of the rainfall simulation experiment and the first time, surface runoff was collected in a trench at the lower end of the irrigation plot. It is a measure of the delay in the surface runoff response and is usually the smaller the larger the final SRC [35].
(4) Initial SRC gradient: gradient of the rising limb of the discharge curve, derived from a fitted linear model based on the records before reaching the maximum curvature. In addition to current runoff, it depends on roughness and the slope of the plot.
(5) Maximum curvature: the maximum change of the gradient of the spline fitted in the discharge curve. It can be interpreted as an indicator for the retention capacity of a site. Small curvature values indicate a low retention capacity and thus rapid runoff processes, e.g. surface runoff due to infiltration excess. Large curvature values indicate runoff processes developing continuously during the experiment, e.g. surface runoff due to saturation excess.

Initial SRC gradient and maximum curvature refer to the change in the discharge coefficient (first and second derivate) and therefore have the unit min^−1^ (initial SRC gradient) and min^−2^ (curvature).

Since the aim of this study is to find out which sites react sensitively to the ASMC, the differences of the SRH parameter values between the first and the repeat experiment are considered for each derived parameter (see Table 4). For the time to runoff, the (usually longer) time measured in the first experiment minus the (usually shorter) time measured in the second experiment was determined. For the other four parameters, the (usually lower) value of the first experiment was subtracted from the (usually higher) value of the second experiment. The result of the calculated differences was thus ≥0 for all parameters. Since third experiments are only available at four sites, they are not included in the calculation of the differences.

**Table 4:** Definition of the SRH parameter differences.

Abbreviation	Definition.
Diff.tot.SRC	Total SRC of experiment 2 minus total SRC of experiment 1
Dff.fin.SRC	Final SRC of experiment 2 minus final SRC of experiment 1
Diff.time	Time to runoff of experiment 1 minus time to runoff of experiment 2
Diff.SRC.grad	Initial SRC gradient of experiment 2 minus initial gradient of experiment 1
Diff.max.curv	Maximum curvature of experiment 2 minus maximum curvature of experiment 1

Soil physical parameters were collected for all rainfall simulation sites using loose soil material and undisturbed soil cores (200 cm³ cylinders) in different depths: 0–10 cm, 11–20 cm, 21–30 cm. For better readability, these depth levels are referred to as 5 cm, 15 cm and 25 cm in the following. By sieving loose soil material, we obtained the grain size proportions for the two classes coarse fragments (>2 mm) and sand (>63 μm-2 mm). An optical light diffraction procedure (Malvern Mastersizer 2000/3000E) was used to analyse the proportion of silt (>2–63 μm) and clay (≤2 μm). For deriving the soil water retention characteristics of the samples from Brixentalbach and Ruggbach catchments, we analyzed (i) saturated undisturbed soil cores with a capillary method [44] as well as with a pressure plate apparatus (330 hPa) and (ii) saturated loose material at different pressure levels (1000 hPa; 3000 hPa; 5000 hPa and 15,000 hPa) [27]. Soil water retention characteristics for Istalanzbach, Kapron and Tanas samples were determined by means of the HYPROP system [45]. The bulk density and content of organic matter were identified gravimetrically, the latter by loss on ignition at 430 °C.

For the representation of the ASMC, we used the relative saturation deficit SD for each depth level x and time t calculated by

$${SD}_{xt}=1-\frac{{SM}_{xt}}{P_{x}}$$

with SM_xt_ = soil moisture at the depth level x [vol%] and time t, P_x_ = porosity at the depth level x [%].

Fig. 3b shows the typical sequence of experiments: The saturation deficit (SDB1) at the beginning of the first experiment is reduced by irrigation (SDA1). Between the irrigation experiments, there is a drying phase (due to percolation and evapotranspiration) and thus a slight increase in the saturation deficit (SDB2), which is reduced again by the following irrigation (SDA2).

## 3 Results

### 3.1 Surface runoff hydrographs

**Fig. 4:**
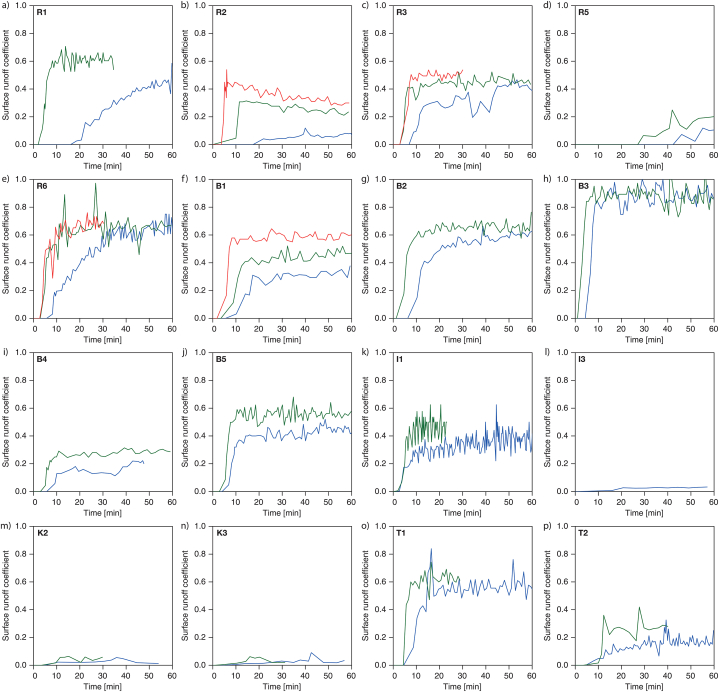
Surface runoff hydrographs of the repeated rainfall simulation experiments for all sites at which surface runoff occurred during the rainfall simulation experiment (60 min). Blue = experiment 1, green = experiment 2, red = experiment 3. Description of the site names see Table 1. At the sites R4, I2, K1 and T3 no surface runoff developed during the experiments. (For interpretation of the references to colour in this figure legend, the reader is referred to the Web version of this article.)

The results of the repeated rainfall simulation experiments are shown in Table 5 and Fig. 4a-p. Usually, the sprinkler system was switched off after 60 min. In rare cases, however, it ran longer in order to further observe particular effects. Therefore, for reasons of better comparability, the results are displayed uniformly for 60 min duration and the falling branch of the surface runoff hydrograph after the end of irrigation is not shown.

**Table 5:** Rainfall simulation experiment results (NDF = not defined).

Site	Total SRC [−]			Final SRC [−]			Time to runoff [min:sec]			Initial SRC gradient [% min^−1^]			Maximum curvature [% min^−2^]		
	1	2	3	1	2	3	1	2	3	1	2	3	1	2	3
R1	0.22	0.53	–	0.45	0.60	–	16:30	02:00	–	1.07	10.93	–	0.04	6.48	–
R2	0.04	0.23	0.33	0.06	0.23	0.27	17:23	10:00	03:40	0.27	3.32	14.04	0.02	0.58	5.94
R3	0.32	0.41	0.42	0.42	0.45	0.52	06:50	02:51	02:50	3.20	8.03	10.80	0.51	2.66	17.38
R4	0.00	0.00	–	0.00	0.00	–	NDF	NDF	–	NDF	NDF	–	NDF	NDF	–
R5	0.03	0.08	–	0.00	0.00	–	42:17	27:00	–	NDF	NDF		NDF	NDF	
R6	0.45	0.59	0.55	0.63	0.67	0.70	05:40	02:53	02:40	3.09	19.44	22.26	0.30	10.89	13.79
B1	0.24	0.38	0.54	0.36	0.50	0.61	05:30	03:30	01:50	1.91	4.34	10.27	0.16	0.78	3.57
B2	0.46	0.60	–	0.61	0.65	–	06:17	01:17	–	3.49	9.68	–	0.39	3.65	–
B3	0.79	0.85	–	0.88	0.89	–	04:20	01:00	–	15.84	16.64	–	4.58	4.73	–
B4	0.13	0.25	–	0.23	0.29	–	05:40	02:57	–	1.85	4.16	–	0.31	2.14	–
B5	0.36	0.50	–	0.47	0.56	–	04:00	02:50	–	4.20	9.79	–	0.86	3.13	–
I1	0.30	0.36	–	0.40	0.47	–	02:39	02:00	–	3.87	4.66	–	0.73	0.92	–
I2	0.01	0.01	–	0.00	0.00	–	22:53	NDF	–	NDF	NDF	–	NDF	NDF	–
I3	0.02	0.02	–	0.00	0.00	–	NDF	09:20	–	NDF	NDF	–	NDF	NDF	–
I4	0.00	0.00	–	0.00	0.00	–	NDF	NDF	–	NDF	NDF	–	NDF	NDF	–
K1	0.01	0.00	–	0.00	0.00	–	37:02	07:00	–	NDF	NDF	–	NDF	NDF	–
K2	0.02	0.03	–	0.00	0.00	–	05:00	03:20	–	NDF	NDF	–	NDF	NDF	–
K3	0.02	0.03	–	0.00	0.00	–	03:02	00:42	–	NDF	NDF	–	NDF	NDF	–
T1	0.49	0.51	–	0.63	0.65	–	04:32	04:22	–	7.85	26.73	–	1.45	18.04	–
T2	0.12	0.19	–	0.19	0.28	–	04:35	03:27	–	0.67	2.27	–	0.03	0.37	–
T3	0.00	0.00	–	0.00	0.00	–	NDF	NDF	–	NDF	NDF	–	NDF	NDF	–

**Table 6:** (a) Saturation deficit values and (b) soil physical parameters for the Pearson correlation analysis in Fig. 8.

(a) Number in … depth.			Abbr	Saturation deficit parameter [−]
5 cm	15 cm	25 cm		
1	9	17	SDB1	saturation deficit before experiment 1
2	10	18	SDA1	saturation deficit after experiment 1
3	11	19	SDB2	saturation deficit before experiment 2
4	12	20	SDA2	saturation deficit after experiment 2
5	13	21	DSDB	saturation deficit before experiment 2 minus saturation deficit before experiment 1
6	14	22	DSDA	saturation deficit after experiment 2 minus saturation deficit after experiment 1
7	15	23	DSD1	saturation deficit after experiment 1 minus saturation deficit before experiment 1
8	16	24	DSD2	saturation deficit after experiment 2 minus saturation deficit before experiment 2

(b) Number in…depth.			Abbr	Soil physical parameter	Size
5 cm	15 cm	25 cm			
25	36	47	CF	coarse fragments [%]	>2 mm
26	37	48	SA	sand [%]	>63 μm-2 mm
27	38	49	SI	silt [%]	>2–63 μm
28	39	50	CL	clay [%]	≤2 μm
29	40	51	XP	coarsest pores [%]	>50 μm
30	41	52	CP	coarse pores [%]	>10–50 μm
31	42	53	MP	medium pores [%]	0.2–10 μm
32	43	54	FP	fine pores [%]	<0.2 μm
33	44	55	PV	porosity [%]	
34	45	56	BD	bulk density [g cm-³]	
35	46	57	OM	organic matter [%]

Depending on the site characteristics and the pre-conditions encountered during the first experiment, the surface runoff hydrographs differ strongly. The partly shorter duration of the experiments explained in section 2.2 due to water supply issues (R1, R3, R6, B4, I, K, T) is noticeable, but does not substantially affect data analysis and interpretation.

**Fig. 5:**
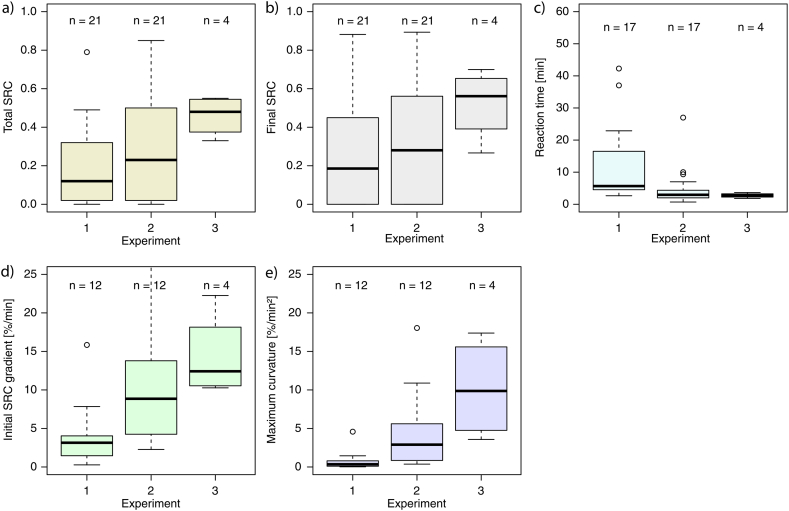
(a) Total surface runoff coefficient (SRC), (b) final SRC, (c) time to runoff, (d) initial SRC gradient and (e) maximum curvature for the first, second and third rainfall simulation experiments.

As expected, the surface runoff coefficients increased from experiment to experiment (Fig. 5a and b) with exception of most of the forest sites. The increase was more pronounced for the total SRC than the final SRC values. The time to runoff decreased (Fig. 5c), the rising branch of the surface runoff hydrograph became steeper (Fig. 5d) and the curvature more concise (Fig. 5e).

Forest sites produced no (R4, I2, K1, T3) or very low surface runoff (R5, I3, K3, T2) with one exception (T2), a black pine forest (Fig. 4p, for discussion see section 4). The properties of forest opening I4 with dwarf shrub cover (blueberry, *Vaccinium myrtillus*) promote infiltration and therefore no surface runoff was produced, analogous to an avalanche corridor surrounded by forest (K2). In contrast to this, the forest opening I1 produced significant surface runoff due to partly topsoil-compaction during logging operations and the hydrophobic needle litter in the lower part of the test area. Significant surface runoff was also observed on the hay meadows (R1, R2, R3, R6) and pastures (B1, B2, B3, B4/B5 and T1), due to topsoil-compacting land use.

The surface runoff hydrographs start with an ascending branch that turns into a more or less horizontal branch. The latter often shows an undulating form, which on the one hand has to do with the measuring method, but on the other hand may also be due to the irregularity of the surface runoff related to its emergence and its flow on the rough surface within the plots. In general, a quasi-steady surface runoff can be observed from a certain moment of the experiment. At a range of sites, however, especially in the first experiment, the surface runoff hydrograph is still rising at the end of the experiment indicating that an equilibrium state in runoff generation has not yet been reached. A special case is R2: There, the surface runoff hydrographs of the second and third experiments are descending. Possible reasons for this unusual surface runoff hydrograph form are discussed in section 4.

### 3.2 Effect of antecedent soil moisture on surface runoff coefficients

**Fig. 6:**
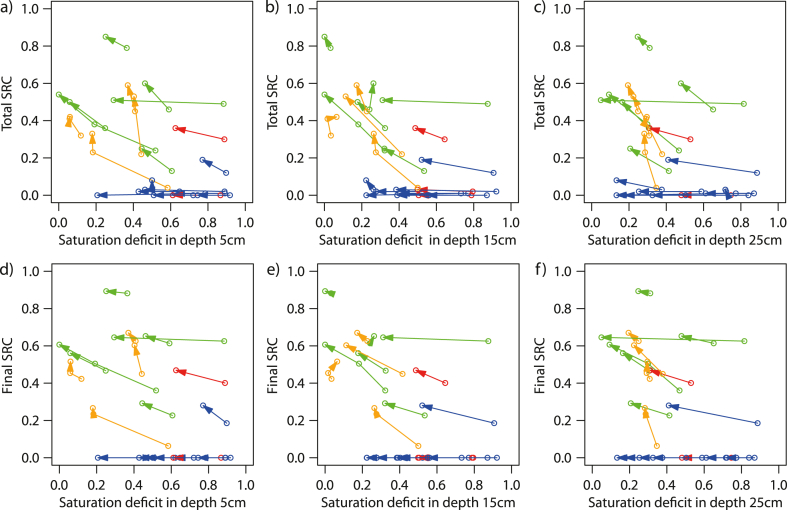
Saturation deficit at the beginning of the respective experiment in (a) 5 cm, (b) 15 cm, (c) 25 cm depth versus total SRC [−]. The vector connects the total/final SRC of the first experiment (vector shaft) with the total/final SRC of the second experiment (vector head). Vector colours: green = pasture sites, orange = hay meadow sites, blue = forest sites, red = openings. The negative saturation deficit values in a few second and third experiments result when the pore volume at the site where the TDR measurement took place is slightly larger than in the soil sample taken. (For interpretation of the references to colour in this figure legend, the reader is referred to the Web version of this article.)

The first experiment at each site took place at the actual soil moisture conditions. In contrast to laboratory conditions, comparable antecedent soil moisture conditions at all sites cannot be produced outdoors due to the different precipitation history and the temporal and logistical framework conditions (availability of the irrigation team, project schedule). The preceding precipitation and evapotranspiration conditions as well as the porosity present at the site control the saturation deficit at the beginning of the first experiment. Fig. 6a–c shows the change of the saturation deficit at the beginning of each experiment as well as the development of the total SRC from the first to the second and – if carried out – to the third experiment. For the forest sites, the saturation deficit is usually high at the beginning of the first experiment due to e.g. interception, tree transpiration and the high fraction of coarser pores (see Fig. 9) and decreases from the first to the second experiment, but the total SRC remains practically zero. Thus, the surface runoff reaction of the forest sites does not depend on the soil moisture conditions. The openings behave similar as forest sites because, despite the removal of forest due to logging (I4) or avalanches (K2), the infiltration-promoting forest soil properties have still been preserved and large parts of the formerly bare forest soil was covered by ground vegetation which developed after the timber harvest. The only opening with significant surface runoff (I1) showed signs of topsoil compaction and a larger part still was merely covered by water repellent spruce litter.

At the hay meadow sites on silt loam (classification according to Ref. [46]) in the Ruggbach catchment, the saturation deficit decreases in the upper two depth levels, but the water hardly reaches the third depth level (25 cm), where the saturation deficit remains constant. The total SRC values of the hay meadow sites always rise in the second experiment, whereby the increase tends to be greater the lower the saturation deficit (Fig. 6). In the pasture areas, which are mostly located in the Brixenbach catchment, the saturation deficit before the first experiment is low to medium. There is a clear increase in total SRC for the second experiment, with two exceptions: At site B3, the total SRC is high (0.79) for the first experiment due to intensive grazing (seasonal compaction of topsoil, loss of soil cover and subsequent silting, accumulation of dead biomass). Thus, the second experiment shows only a slightly increased total SRC. At site T1, characterized by a *Nardus stricta* L. (matgrass) heath situated between the pine forest (T2) at the one side and groups of bushes at the other side, there is a very clear reduction in the saturation deficit, which was very high at the beginning, but the total SRC remains around 0.5. The high SRC is primarily a result of the wetting inhibiting effect of the dead leaf sheaths and the root felt in the uppermost soil layer of the matgrass [35,47], and not depending on the ASMC. The picture of the final SRC vectors is quite similar to that given by the total SRC vectors (Fig. 6d-f).

**Fig. 7:**
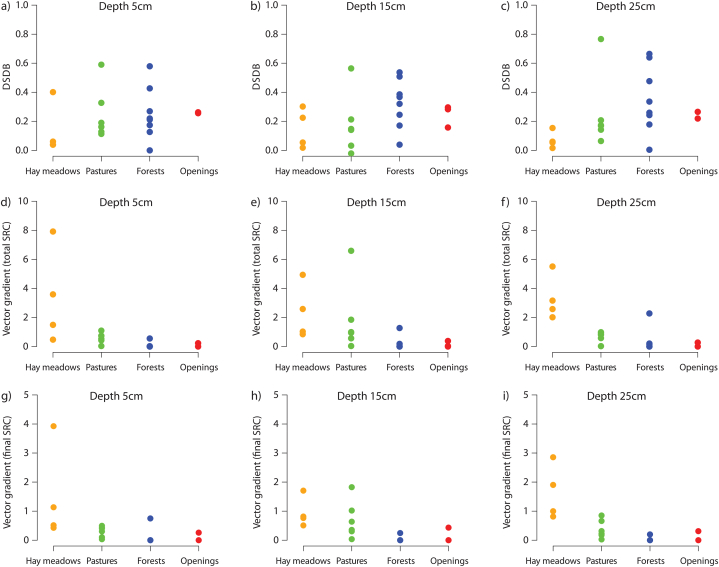
(a)–(c) x-component of the vectors from Fig. 6 (DSDB = saturation deficit before experiment 2 minus saturation deficit before experiment 1) representing the water retention. (d) and (e) Gradients of the vectors shown in Fig. 6 referring to (d)–(f) the total SRC, (g)–(i) the final SRC. The vector gradients indicate the effect of the saturation deficit at the beginning of the experiment on the surface runoff production.

**Fig. 8:**
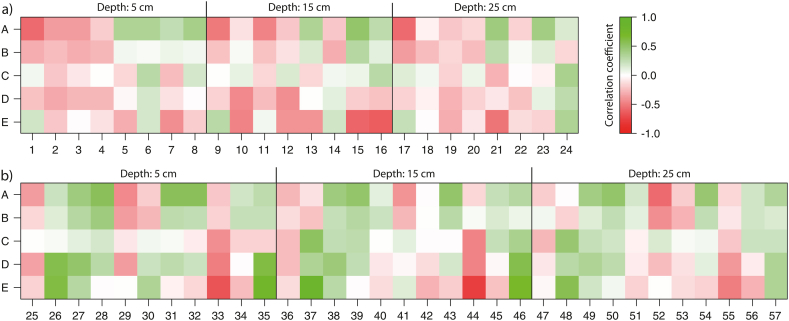
Pearson correlations coefficients between (a) saturation deficit values (explanation of the numbers see Table 6a)/(b) the soil physical parameters (see Table 6b) and the differences of the SRH parameters (value of experiment 2 minus value of experiment 1): difference of (A) total SRC, (B) final SRC, (C) time to runoff, (D) initial SRC gradient, (E) maximum curvature.

Fig. 7 gives a summary of the statements above: The gradients of the vectors shown in Fig. 6 indicate the effect of the saturation deficit at the beginning of the experiment on the surface runoff production. The x-components of the vectors show the water retention caused by the preceding experiment*.* The hay meadow sites reacted with a strong increase in surface runoff to changed saturation deficits. However, it should be borne in mind that the hay meadow sites are all located in the same area, namely in the Ruggbach catchment (fine-grained molasse sediments). The investigated pasture sites showed a smaller but visible increase of surface runoff in the repeated experiment. The forest sites had the highest water retention values. There are only minor differences between the vectors of the SRC and the final SRC. Looking at the differences of the derived SRH parameters (total SRC, final SRC, time to runoff, initial SRC gradient and maximum curvature) between the two experiments, we investigated their Pearson's correlation with different saturation deficit parameters (see Fig. 8a and Table 5a).

**Fig. 9:**
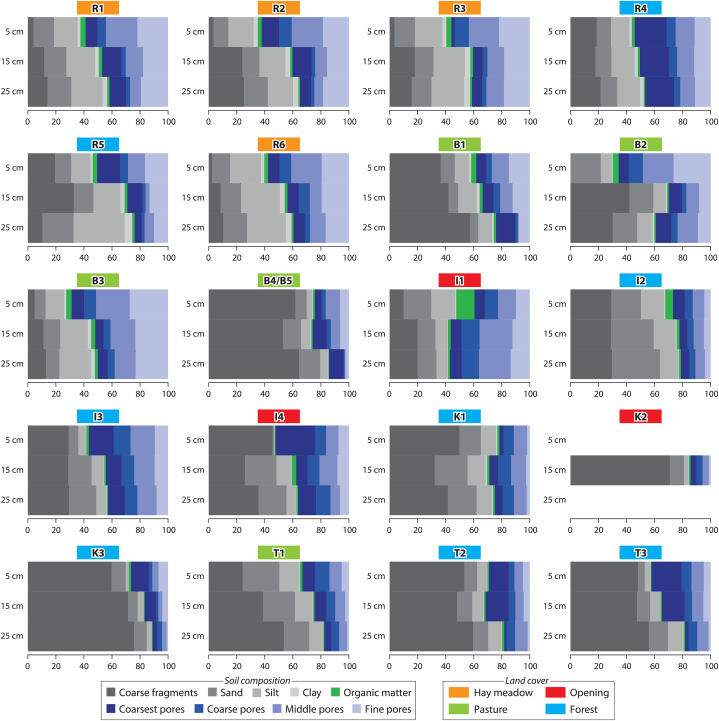
Coarse fragments (>2 mm), fine soil (sand >63 μm-2 mm, silt > 2–63 μm, clay ≤2 μm) and pores (coarsest >50 μm, coarse >10–50 μm, middle 0.2–10 μm, fine <0.2 μm) of the investigated sites in the depth levels of 5, 15 and 25 cm [in vol%]. Description of the site names see Table 1. At site K2, no undisturbed samples could be taken at depths of 5 and 25 cm due to the high content of coarse material.

The smaller the saturation deficit before the first experiment the greater the difference in total SRC, i.e., the less infiltration occurred in the second experiment, leading to high surface runoff (line A in Fig. 8b). Similar applies to the difference of the final SRC, but with a lower correlation (higher negative correlation coefficient value). The correlations of the saturation deficits with the other SRH parameters are weaker and often would even have a smaller amount if all outliers were eliminated.

### 3.3 Role of the soil physical characteristics

Both the surface runoff response and the soil moisture dynamics depend on the soil water retention characteristics. The investigated sites show a great variety in terms of the proportion of coarse fragments (>2 mm), fines (sand >63 μm–2 mm, silt >2–63 μm, clay ≤2 μm) and the porosity (coarsest pores >50 μm, coarse pores 10–50 μm, middle pores 0.2–10 μm, fine pores <0.2 μm) in the depth levels 5 cm, 15 cm and 25 cm (Fig. 9). The sites in the Ruggbach catchment (hay meadows) stand out due to their comparatively low content of coarse fragments. The question of whether the SRH parameter differences are related to soil physical parameters was investigated with another Pearson's correlation analysis. It reveals low to medium correlation coefficients between the SRH parameter differences and the soil physical parameters (Fig. 7b). The greater the fine pore fraction is at all three depth levels as well as the medium pore fraction and clay content at 5 cm depth, the greater the increase in total SRC from the first to the second experiment. These three soil physical parameters also correlate positively with each other.

**Fig. 10:**
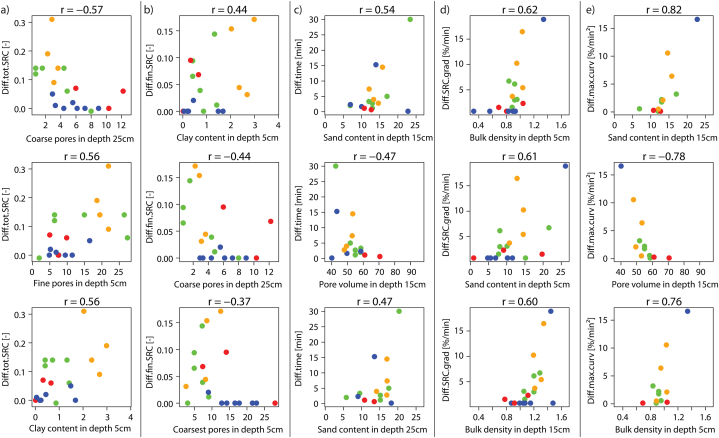
The strongest three correlations for each hydrograph parameter difference (see Table 4) with soil physical parameters. Dot colours: green = pastures, orange = hay meadows, blue = forests, red = openings. (For interpretation of the references to colour in this figure legend, the reader is referred to the Web version of this article.)

The orange dots in Fig. 10a show that the hay meadow sites, which are all situated in the Ruggbach catchment, have homogeneous fine pore distributions in 5 cm depth (as well as coarse pore distribution in 25 cm depth), but a large range in the difference of the total SRC. Conversely, the pasture sites (green dots) have a larger range in the fine pore distribution, but the difference between the total SRC of the first and the second experiment has a quite narrow range from 0.06 to 0.14 (with one exception – the difference of the total SRC of the mat grass dominated site T1 at the edge of a forest is 0.02). The forest sites show even lower total SRC differences with also a relatively wide range of pore distribution.

The correlations between soil physical parameters and the difference in final SRC values (Fig. 10b) are generally lower compared to the difference of the total SRC values. The correlation plots also show a diffuse picture.

The difference of the time to runoff between the first and the second experiment has the largest (positive) correlation coefficients with sand content and porosity. The higher the sand content, especially in the lower two depth levels, the more the (longer) time to runoff of the first experiment differs from the (shorter) time to runoff of the second experiment (Fig. 10c and line C in Fig. 8a). The longest difference in times to surface runoff (approx. 30 min) occurs at a forest site (K1) with a very low SRC and has thus only little informative value.

The change in the initial SRC gradient from the first to the second experiment is the stronger the higher the bulk density in 5 and 15 cm depth (Fig. 10d and line D in Fig. 8a). The correlation coefficients are somewhat higher for the difference in maximum curvature, which is mainly due to the smaller data set (no curvature at sites without surface runoff analysable). The curvature increases in the second experiment compared to the first, the smaller the pore volume (especially at a depth of 15 cm) and the greater sand content (15 cm depth) and the bulk density (5 cm depth) (see line E in Fig. 7, Fig. 10e).

**Fig. 11:**
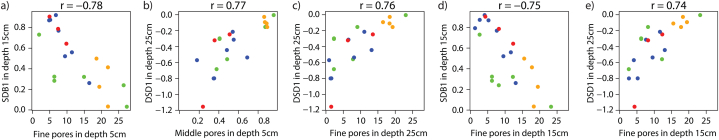
Strongest correlations between soil physical parameters (Abbreviations see Table 6a) and the saturation deficit before the first experiment in 15 cm depth (SDB1_15 cm) (a), (d)) or the difference of the saturation deficit before and after the first experiment in 25 cm (DSD1_25 cm). Dot colours: green = pastures, orange = hay meadows, blue = forests, red = openings. (For interpretation of the references to colour in this figure legend, the reader is referred to the Web version of this article.)

The saturation deficits encountered at the begin of each first experiment correlate with the site characteristics even stronger than the SRH parameter differences do (Fig. 11). The saturation deficit at 15 cm depth shows a strong negative correlation with the fine pore fraction in 5 and 15 cm (Fig. 11a, d). Strong correlations (0.7) can also be observed between the difference of the saturation deficit before and after the first experiment and the medium pore fraction in 5 cm depth (Fig. 11b) as well as the fine pore fraction in 25 cm (Fig. 11c) and 15 cm depth (Fig. 11e).

## 4 Discussion

### 4.1 Role of the fine pore fraction

As shown, the fine pore fraction at 5 cm depth correlates both with the difference in total SRC (positively) and (negatively, but even more strongly) with the saturation deficit at the beginning of the first experiments. However, the soil water content measured at the beginning of the first experiment was greater than the fine pore fraction at all 20 sites. So the fine pores were in any case filled with water at the beginning of the first experiment, and in most cases a large proportion of the medium pores was also saturated, which is usually the case in moist climates. The fine pore fraction is therefore not a proxy of the current system state at the beginning of the first experiment, but a site characteristic, with the help of which, firstly, a fair statement (Pearson correlation coefficient 0.56) can be made about how strongly the total SRC changes from the first to the second experiment with reduced saturation deficit, i.e., whether the surface runoff development reacts sensitively to soil moisture changes. Secondly, a soil's fine pore fraction allows a statement about the saturation deficit that is probably to be expected at a site at the beginning of the first experiment (Pearson correlation coefficient −0.78). Even if this depends on the antecedent weather conditions (precipitation, air temperature), the porosity and the pore size distribution determine the possible range of the saturation deficit. With this regard, the proportion of fine pores seems to be a controlling parameter.

The extent of the shortening of the time to (surface) runoff, the steepening of the initial SRC gradient and the increase in the maximum curvature show less clear dependencies on the soil physical parameters investigated.

### 4.2 Land use associated patterns

Fig. 6, Fig. 10, Fig. 11 reveal certain patterns associated with land use. That land use plays a significant role is shown by the experimental pairs B4 and B5 at an intensively used pasture site in the Brixenbach catchment [36]. It was irrigated twice in May 2012 before the start of the grazing season (B4). After the end of the grazing season in September 2012, the rainfall simulation was repeated on exactly the same plot (B5). The results show an increase in total SRC from the first experiment (at current soil moisture conditions) to the second experiment (at wet conditions) by 0.12–0.14, whereas the total SRC value after the grazing season increased by about 0.22–0.25 when pairing the experiments with comparable soil moisture conditions (Fig. 4i and j). The measured effect of grazing thus strongly exceeds the measured effect of the soil moisture status related to a jump from more or less average to high ASMC. The reasons lie in the composition of the biomass, seasonal topsoil compaction, loss of soil cover and subsequent silting, and hydrophobic effects due to the accumulation of dead biomass with the continuation of the grazing season [48]. In Tanas, it could be shown that the composition of the forest also plays a major role. Forest soils are usually considered to have favourable infiltration properties [[49], [50], [51]]. The black pine forest afforested in the 1950s to reduce erosion processes yielded a total SRC value of 0.12 in the first irrigation experiment, and a value of 0.19 in the second experiment simulating very moist conditions (T2, Fig. 4p). Rainfall simulation experiments in Tanas took place after months without major rainfall [38]. Therefore, the hydrophobic effect of the pine litter and the dried organic matter in the uppermost soil layers may have been particularly high. Staffler et al. [52] describe the humus form as a xeromorphic moder, as the needle litter of pines contains considerable shares of resins, waxes or aromatic oils [13]. Heinkele et al. [53] observed pronounced hydrophobic properties already at water contents in the humic layer far above the permanent wilting point. At T2, preferential flow paths were already formed on the surface during the first experiment.

Although the black pines are very well suited to the dry site conditions on the south-facing slope in this inner-Alpine dry valley, they show increasing loss of species, a high risk of forest fires, decrease of stand stability and mass reproduction of pathogens (pine processionary moth). Therefore, stand conversion of black pine into hardwood-rich stands was started around the turn of the millennium [54]. In such young stands, which were fenced off to protect them from browsing by game, surface runoff could not be generated by either the first or the repeated rainfall simulation experiment (T3) [37].

The influence of land use on surface runoff generation is also validated in numerous other studies [16,18,19,22,23,35]. Land use in turn has an impact on soil properties, e.g. soil pore distribution and bulk density [55,56].

Site R2 shows an exceptional behaviour: During the second and third experiment, the maximum surface runoff is reached at the end of the ascending branch of the surface runoff hydrograph immediately, then the surface runoff hydrograph descends again to a certain extent (Fig. 4b). This behaviour could not be observed in any other irrigation experiment of this study. A look at the soil physics data shows that site R2 has the highest clay content of all sites. Possibly the pore wall stability was reduced by the saturation of the soil during the previous rainfall simulation experiment, which led to a short-term sealing of the pore channels when irrigation resumed (miscible displacement) [57]. However, the latter may have been flushed free again in the course of further irrigation, leading to the decreasing surface runoff coefficient.

### 4.3 Reaction types

According to the high rainfall intensity applied, the runoff mechanism is predominantly infiltration excess. This is especially true for the pasture sites with their typical topsoil compaction and the reduced vegetation cover. However, the low saturation deficits occurring at some sites (see Fig. 6) also indicate the occurrence of saturation excess.

In summary, based on the total SRC values of the first experiment and the extent of change to the second experiment, the sites can be divided into four types:

(1) Sites where the surface runoff response does hardly or not change in the second experiment:
  Type 1.1: Sites with very low surface runoff response: These are the investigated forest sites (with exception of T2), which all have favourable infiltration and percolation properties (R4, R5, I2, I3, K1, K3, T3). Openings whose soil properties are still favourable for infiltration despite the change in vegetation cover (I4, K2) are also included. High percolation performance is achieved through high organic content in the mineral soil as well as a high proportion of macropores. The pore volume can be used quickly including deeper layers for water retention.
  Type 1.2: Sites with very high surface runoff response. This type is represented by B3 in the present study. It is a very intensively used pasture on a Stagnosol, which already had a very high total SRC (0.79) in the first experiment, meaning that only a small amount of water infiltrated. Thus, the amount of surface runoff in the second experiment did not increase much. Saturation areas behave similarly. They produce high surface runoff coefficients but do not reach 1 even at high ASMC [58].
(2) Sites where the surface runoff response changes after initial irrigation:
  Type 2.1: Sites with medium surface runoff response and a fair increase in total SRC <0.1. These include openings I1 and T1 as well as R3, a hay meadow site in direct vicinity of a forest which is thus influenced by spreading roots. The pine forest T2 also belongs to this type with its runoff behaviour primarily attributed to the formation of hydrophobic effects after drought. These are not overcome even after the 1-h pre-irrigation.
  Type 2.2: Sites with a significant increase in total SRC >0.1. These include hay meadow and pasture sites R2, R6, B1, B2, B4/5 and, as an outlier, R1 with an increase in total SRC of 0.31. These sites are characterized by adequate infiltration capacity under medium and dry conditions. At high saturation, the rapidly available pore spaces of the mineral soil are not available and thus the retention capacity is limited.

With regard to the other SRH parameters investigated (difference of final SRCs, times to runoff, initial SRC gradients and maximum curvatures), however, these four types do not differ significantly. The total SRC integrates all investigated parameters, these behave with increasing soil moisture in runoff enhancing direction (Fig. 5).

Predicting in advance how a site will react in terms of surface runoff remains still difficult after the completion of our study. As mentioned, the ASMC was not controllable in the first experiment – this is a disadvantage of field studies compared to laboratory experiments. The conditions were mostly average without extreme antecedent rainfalls or dry periods (apart from dry conditions in Istalanzbach catchment and Tanas). However, longer dry periods are especially typical for the sites in Tanas situated at a south-exposed slope in an inner Alpine dry valley. After 100 mm h^−1^ were applied in the first experiment, the second experiment took place at those pre-conditions, which are site-specific for very moist situations and depend, e.g. on the total SRC which controls the amount of infiltrating water and the drainage characteristics of the site. The differences in the SRH parameters between the experiments, however, naturally depend on the level of the values in the first experiment, which are subject to a certain degree of uncertainty and should therefore be considered more in its order of magnitude, less related to the exact value. Despite this limitation, we believe that the SRH parameter differences represent the site responses well.

### 4.4 Macropores

As shown, there are moderate correlations between the differences of the SRH parameters and soil physical properties, especially the (fine) pore volumes. However, a large proportion remains unexplained in the linear analysis. Threshold-controlled reactions are emerging, but the data set is too small to determine them. This is to our opinion mainly related to macropore network connectivity, as already noted by Ries et al. [21]. This may also be the reason why the clusters of sites based on principal component analysis of soil physical data did not differ significantly in terms of SRH parameter differences and are therefore not shown here.

Macropore flow plays a major role at the unploughed hay meadow and pasture sites and therefore also controls the infiltration capacity [59,60]. Macropore flow initiated at the soil surface is characterized by a very uneven distribution of inflow to the macropores: Only a few macropores receive the largest share of water, while most macropores typically remain almost dry [61]. In forest soils with near-surface macropores (root tubes, fissures, shrinkage cracks), mean flow velocities of 500 m d^−1^ and more are possible [62,63].

Macropore flow was made visible with the help of dye tracers in the Brixenbach catchment [27,64]. However, measuring macropore network connectivity is very difficult. The determination of saturated hydraulic conductivity in the laboratory using undisturbed soil samples shows very large uncertainties if the sample contains macropores and many coarse fragments as Alpine soils do. In this case, it is very difficult to extract an undisturbed soil core. In addition, the size of a soil cylinder is too small to represent macropore network connectivity on a plot scale. The use of x-ray tomography to determine macropore network connectivity as presented by Jarvis et al. [65] or the measurement of macropore flow velocity as a proxy [66] was also not possible in this study.

## 5 Conclusions

In order to understand for which Alpine sites the surface runoff response depends to what extent on antecedent soil moisture conditions, soil characteristics and land use, 20 sites in five areas were analyzed by means of repeated rainfall simulation experiments (40–80 m^2^, 1 h, 100 mm h^−1^).

As expected, on the plots, where surface runoff occurred, the second and (if executed) third experiments showed a higher surface runoff coefficient, a shorter time to surface runoff, a steeper gradient of the rising branch of the surface runoff hydrograph and a stronger curvature at the end of the rising branch. We drew vectors connecting the point defined by saturation deficit at the beginning of the experiment and SRC obtained in the experiment with the respective values from the following experiment. They can be used to show how much the surface runoff changes due to the increased antecedent soil moisture. The hay meadow sites reacted with a strong increase in surface runoff to reduced saturation deficits, the pasture sites showed a smaller but visible response. The forest sites had the highest water retention capacities.

The change in the surface runoff response is a function of the saturation deficit at the beginning of the first experiment (r = −0.58). The soil physical parameters, among others the fine pore fraction (r = 0.56), correlate with the difference of the total SRC between the first and the second experiment. The fine pore fraction has also a high correlation (r = −0.78) with the saturation deficit at the beginning of the first experiment, although it was saturated before and during all experiments, it is therefore not a proxy for the state of the system.

(Non-quantifiable) Land use effects, which in turn influence the soil physical parameters, especially the porosity and bulk density, play an important role in explaining how the surface runoff response in the repeated rainfall simulation experiment differs from the initial experiment. Depending on land use and soil characteristics, the sites could be divided into four types in terms of surface runoff response and the increase in total surface runoff coefficient in the second rainfall simulation experiment. Sites were found to have different sensitivities to moisture condition. The highest sensitivity is shown by sites with medium percolation capacity and sufficient soil storage capacity. In contrast, sites characterized by very high infiltration and percolation capacity and sites with very limited infiltration capacity are less sensitive.

However, there is still a great need for research in the recording of important parameters in the field, e.g. macropore network connectivity or interaction between soil storage capacity and macropore connectivity.

## Author contribution statement

Gertraud Meißl: Conceived and designed the experiments; Analyzed and interpreted the data; Contributed reagents, materials, analysis tools or data; Wrote the paper. Klaus Klebinder; Gerhard Markart: Conceived and designed the experiments; Performed the experiments; Analyzed and interpreted the data; Contributed reagents, materials, analysis tools or data; Wrote the paper. Thomas Zieher: Analyzed and interpreted the data; Contributed reagents, materials, analysis tools or data; Wrote the paper. Veronika Lechner: Conceived and designed the experiments; Performed the experiments; Analyzed and interpreted the data; Contributed reagents, materials, analysis tools or data. Bernhard Kohl: Conceived and designed the experiments; Analyzed and interpreted the data; Contributed reagents, materials, analysis tools or data.

## Data availability statement

Data will be made available on request.

## Declaration of competing interest

The authors declare that they have no known competing financial interests or personal relationships that could have appeared to influence the work reported in this paper.
